# Sialylation profile and Siglec-E expression across tissues in the B16OVA melanoma mouse model

**DOI:** 10.1093/glycob/cwag041

**Published:** 2026-06-01

**Authors:** Magali Coccimiglio, Tao Zhang, Katarzyna Olesek, Laura Goossens-Kruijssen, Noortje de Haan, Fabrizio Chiodo, Yvette van Kooyk

**Affiliations:** Department of Molecular Cell Biology & Immunology, Amsterdam UMC Location VUmc, De Boelelaan 1108, 1081 HZ, 1007 MB, Amsterdam, The Netherlands; Cancer Center Amsterdam, De Boelelaan 1118, 1081 HV, Amsterdam, The Netherlands; Amsterdam institute for Immunology and Infectious diseases, Amsterdam, The Netherlands; Center for Proteomics and Metabolomics, Leiden University Medical Center, Room P-01-064, Building 1 (route 920), 2333 ZA, Leiden, The Netherlands; Department of Molecular Cell Biology & Immunology, Amsterdam UMC Location VUmc, De Boelelaan 1108, 1081 HZ, 1007 MB, Amsterdam, The Netherlands; Cancer Center Amsterdam, De Boelelaan 1118, 1081 HV, Amsterdam, The Netherlands; Amsterdam institute for Immunology and Infectious diseases, Amsterdam, The Netherlands; Department of Molecular Cell Biology & Immunology, Amsterdam UMC Location VUmc, De Boelelaan 1108, 1081 HZ, 1007 MB, Amsterdam, The Netherlands; Cancer Center Amsterdam, De Boelelaan 1118, 1081 HV, Amsterdam, The Netherlands; Amsterdam institute for Immunology and Infectious diseases, Amsterdam, The Netherlands; Center for Proteomics and Metabolomics, Leiden University Medical Center, Room P-01-064, Building 1 (route 920), 2333 ZA, Leiden, The Netherlands; Italian National Research Council, Institute of Biomolecular Chemistry, Via Campi Flegrei, 34, 80078, Pozzuoli, Naples, Italy; Department of Molecular Cell Biology & Immunology, Amsterdam UMC Location VUmc, De Boelelaan 1108, 1081 HZ, 1007 MB, Amsterdam, The Netherlands; Cancer Center Amsterdam, De Boelelaan 1118, 1081 HV, Amsterdam, The Netherlands; Amsterdam institute for Immunology and Infectious diseases, Amsterdam, The Netherlands

**Keywords:** immune suppression, melanoma, sialylation, Siglec-E, T cells

## Abstract

Increased sialylation of tumour cells, which favours tumour growth and immune evasion, has been described using in vitro and in vivo models, leading to the first in-human clinical trial targeting sialylation in cancer. One important limitation is that the biology of sialic acids and their receptors (Siglecs), which have been considered as immune checkpoints, is different between mice and human. Hence, it is crucial to fully describe and investigate sialic acids and Siglecs expression in animal models, to define their advantages and limitations. Here, we determined the sialylation profile of the widely-used B16OVA melanoma mouse model using flow cytometry and glycomics. B16OVA cells express Siglec-E-binding sialoglycans, therefore we explored Siglec-E expression across various tissues from tumour-bearing mice. We identified that Siglec-E is expressed on myeloid cells and CD8^+^ T cells in the tumour microenvironment. However, in spleen, blood and tumour-draining lymph nodes not only CD8^+^ but also CD4^+^ T cells expressed Siglec-E. Interestingly, Siglec-E^+^ and Siglec-E^−^ T cells presented distinct expression of PD-1 across tissues, suggesting different regulation mechanisms for the expression of these immune checkpoints. Our work provides an investigation of the sialoglycans on B16OVA cells and the expression of their receptor Siglec-E across tissues, which is of importance for future therapeutic studies targeting the sialic acids-Siglec axis, especially in combination with anti-PD-1 therapies.

## Introduction

Tumour cells express glycan structures that differ from healthy cells, which overall constitute the tumour glyco-code (Rodriguez et al. 2018). One of the hallmarks of tumour-related glycan changes is the over-expression of sialic acids, or hypersialylation, which is found across cancer types (Rodriguez et al. 2024; Rodriguez et al. 2018; Stanczak et al. 2022; van Vliet and van Kooyk 2025). Immune cells express receptors for sialic acids called Siglecs (sialic acid-binding immunoglobulin-like lectins), which dampen immune responses upon engagement with their ligands, as they contain inhibitory intracellular motifs similar to the immune checkpoint PD-1 (Stanczak and Laubli 2023). Thus, the sialic acid-Siglec axis has been described as a novel immune checkpoint to target for anti-cancer therapies, especially in combination with current immune checkpoint blockade (ICB) therapies, such as anti-PD-1 antibodies (Mantuano and Laubli 2024).

Over the past decades, the study of the sialic acids-Siglec axis progressed from in vitro to in vivo models, giving rise to different strategies to reduce sialylation in tumours (Bull et al. 2018; Gray et al. 2020; Stanczak et al. 2022). Although glycosylation is a conserved process across mammals, it exhibits species-specific variations (Angata and Varki 2023). The term sialic acids refers to a variety of structures, among which N-acetylneuraminic acid (Neu5Ac) and N-glycolylneuraminic acid (Neu5Gc) are the predominant in mammals. Mice express both Neu5Gc and Neu5Ac, while humans are deficient in Neu5Gc (Lewis et al. 2022). Siglec receptors are divided into two groups according to their conservation among species. Siglecs-1,-2,-4 and − 15 are conserved, while the other Siglecs (CD33-related Siglecs) are rapidly evolving and differ between human and mice. Among these, Siglecs-7 and -9 gained importance in the cancer immunology field, as tumour cells contain ligands for them causing suppression of NK cells, T cells and macrophages (Jandus et al. 2014; Beatson et al. 2016; Haas et al. 2019; Rodriguez et al. 2021; Wen et al. 2024). Although there are no clear orthologues of these Siglecs in mice, Siglec-E is considered the functional paralogue of Siglec-9 (Laubli and Varki 2020). Thus, much work studying the sialic acids-Siglec axis in vivo using mouse models focused on Siglec-E, showing that it modulates T cells, macrophages and myeloid-derived suppressor cells activity in the tumour microenvironment (TME) (Stanczak et al. 2018; Ibarlucea-Benitez et al. 2021; Stanczak et al. 2022; Wieboldt et al. 2024).

The expression of both Siglecs and glycans varies in mice and humans, not only across healthy tissues but also among cancerous ones (Stanczak et al. 2018; Haas et al. 2019; Lee et al. 2020; Mantuano and Laubli 2024; Wu et al. 2026). Consequently, it is crucial to describe animal tumour models in order to improve the translatability of research outcomes from mice to humans, and to expose limitations and advantages of using mouse models to target sialylation. Here, we characterized the sialoglycans of B16OVA, a widely-used melanoma mouse model, and their ability to bind Siglec-E. We determined the expression of Siglec-E on immune cells in vivo in B16OVA tumours, as well as splenocytes, blood and lymph nodes from tumour-bearing mice, which are the tissues that contain the immune repertoire that provides anti-tumour immunity.

## Results

### B16OVA cells contain sialoglycans that serve as Siglec-E ligands

**Figure 1:**
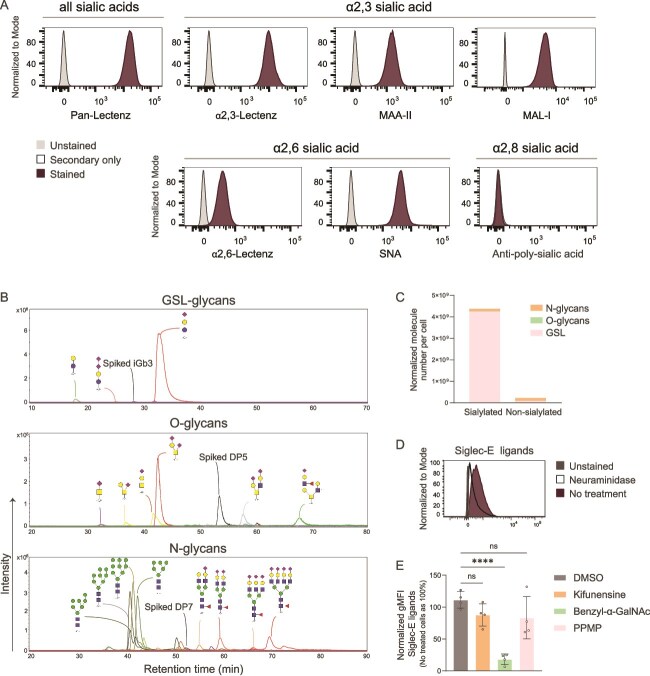
B16OVA cells contain sialoglycans that serve as Siglec-E ligands. A) Histograms showing expression of sialic acids on B16OVA cells determined by flow cytometry. MAA-II = *Maackia amurensis* lectin II, MAL-I = *M. amurensis* lectin I, SNA = *Sambucus nigra* lectin. B) Profiles of glycosphingolipids (GSL)-glycans, O-glycans and N-glycans on B16OVA cells determined by mass spectrometry. C) Normalized abundance of sialylated and non-sialylated GSL-, O- and N-glycans per cell in B16OVA cells. Data from three technical replicates. D) Siglec-E ligands on B16OVA cells determined by flow cytometry (Siglec-E fc binding) after treatment with neuraminidase or no treatment. Data representative of three independent experiments. E) Siglec-E ligands on B16OVA cells determined by flow cytometry after treatment with dimethyl sulfoxide (DMSO), kifunensine, benzyl-α-GalNAc or D-threo-PPMP (PPMP). Data shown as mean ± s.d. of the normalized geometric mean fluorescence intensity (gMFI) over the no treatment condition, from four independent experiments. One-way ANOVA with Dunnett's multiple comparisons test. Ns = *P* < 0.05, ^****^ = *P* < 0.0001.

The sialylation profile of the B16OVA cell line was determined using plant lectins and Lectenz® to detect all sialic acids (Neu5Ac), or specifically α2,3- and α2,6-linked Neu5Ac. We used an anti-poly-sialic acid antibody to detect α2,8-linked Neu5Ac. The B16OVA cell line contains α2,3- and α2,6-linked sialic acids, while α2,8-sialylation was non-detectable (Fig. 1A). We further characterized this cell line with mass spectrometry-based glycomics, finding that sialylated glycosphingolipid (GSL) glycans constituted the largest fraction of the total sialylated glycome, followed by N- and O-glycans (Fig. 1B and C). Although we found low amounts of Neu5Gc-containing GSLs, most sialoglycans in the B16OVA cell line contained Neu5Ac (Supplementary Tables S1-S3). On the GSL-glycans, sialic acids were predominantly found in α2,3-linkage (99.3%), while on N- and O-glycans both α2,3- and α2,6-Neu5Ac were present in a more equal abundance (O-glycans:61.1% α2,3, 38.9% α2,6; N-glycans: not quantified; Fig. 1B, Supplementary Tables S1 and S2).

As Siglec-E is an important mediator of immune cell suppression in tumours (Stanczak et al. 2022), we aimed to determine if the sialylated glycans on B16OVA bind Siglec-E. Using flow cytometry, we found that B16OVA sialoglycans serve as ligands for Siglec-E (Fig. 1D). After treatment with metabolic inhibitors of N-glycosylation, O-glycosylation or GSLs synthesis, we found that those Siglec-E ligands were mostly on O-glycans (Fig. 1E).

### Siglec-E expression in the tumour immune microenvironment

**Figure 2:**
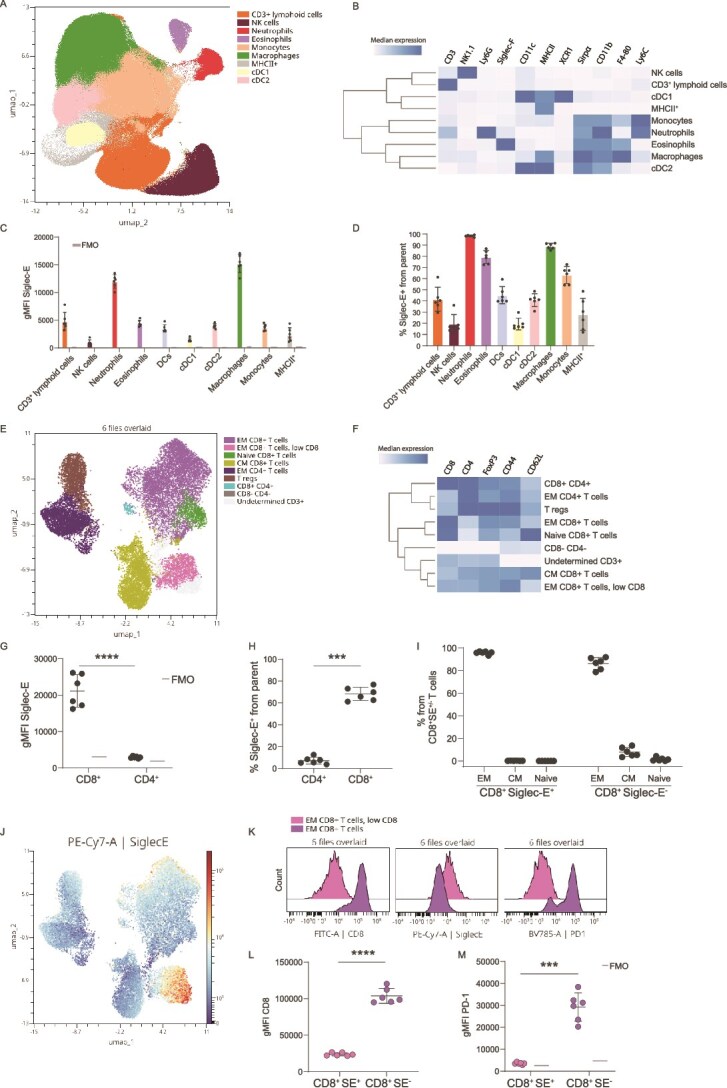
Siglec-E expression in vivo in the tumour immune microenvironment. A) UMAP plot showing main immune cell populations in subcutaneous B16OVA tumours, coloured by cell type. B) Heatmap showing expression of markers in the cell clusters from a. C,D) expression of Siglec-E on the immune cell populations from a as geometric mean fluorescence intensity (gMFI) (C) or percentage positive from each cell population (D). E) UMAP plot showing T cell populations in subcutaneous B16OVA tumours, coloured by cell type. F) Heatmap showing expression of markers in the cell clusters from E. G,H) expression of Siglec-E on CD8^+^ and CD4^+^ T cells as gMFI (G) or percentage positive from each T cell population (H). I) Percentage of effector memory (EM), central memory (CM) or naive CD8^+^ T cells from either Siglec-E^+^ or Siglec-E^−^ CD8^+^ T cells. J) UMAP plot coloured based on Siglec-E expression. K) Histograms showing expression of CD8, Siglec-E and PD-1 on EM CD8^+^ T cell clusters from E. L,M) expression of CD8 (L) and PD-1 (M) on Siglec-E^+^ or Siglec-E^−^ CD8^+^ T cells as gMFI. Data shown as mean ± s.d. from 6 mice. Paired *t-*test for statistical analysis. Ns = *P* > 0.05, ^***^ = *P* < 0.001, ^****^ = *P* < 0.0001. FMO = fluorescence minus one.

We then aimed to characterize the expression of Siglec-E in vivo in B16OVA subcutaneous tumours, to identify immune cells that could potentially interact with the sialoglycans of B16OVA. We could identify the main immune cell populations using unsupervised clustering and manual gating (Fig. 2A and B, Supplementary Fig. S1A-C). Among the myeloid cells, Siglec-E was mostly expressed by macrophages and neutrophils (Fig. 2C and D). We also identified Siglec-E expression on 40% of the tumour-infiltrating CD3^+^ lymphoid cells (Fig. 2C and D).

To identify which CD3^+^ T cell subsets expressed Siglec-E, we performed both unsupervised clustering and manual gating (Fig. 2E and F, Supplementary Fig. S1D-F). B16OVA tumours contained 20% of CD8^+^ T cells and 5% of CD4^+^ T cells (Supplementary Fig. S1E). CD8^+^ T cells expressed high levels of Siglec-E, while almost no expression was found on CD4^+^ T cells (Fig. 2G and H). Siglec-E^+^CD8^+^ T cells were effector memory (EM), while Siglec-E^−^CD8^+^ T cells were mostly effector memory but also a small proportion (around 10%) were central memory (CM) T cells (Fig. 2I, Supplementary Fig. S1F)). Siglec-E was mostly expressed in one of the two clusters identified as EM CD8^+^ T cells (Fig. 2E and J). These two clusters differed on the expression of CD8, PD-1 and Siglec-E. The population with higher Siglec-E expressed less CD8 and PD-1, indicating ongoing activation (Fig. 2K). We could confirm these differences by manual gating, observing that Siglec-E^+^CD8^+^ T cells expressed lower levels of CD8 and almost no PD-1, while the contrary was observed on their Siglec-E^−^ counterparts (Fig. 2L and M). Altogether, B16OVA tumours contain Siglec-E-expressing myeloid and lymphoid cells. Siglec-E is expressed on CD8^+^ but not CD4^+^ tumour-infiltrating T cells, and Siglec-E expression on CD8^+^ T cells relates to their memory phenotype and activation status.

### Siglec-E expression on T cells across tissues from tumour-bearing mice

**Figure 3:**
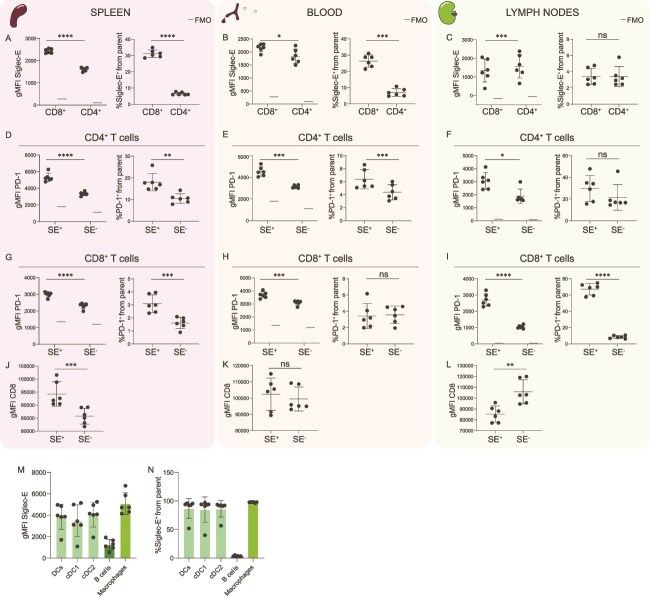
Siglec-E on T cells across tissues and APCs from tdLNs. A-C) expression of Siglec-E on CD4^+^ and CD8^+^ T cells in spleen (A), blood (B) and tdLNs (C) as geometric mean fluorescence intensity (gMFI) or percentage positive from each T cell population. D-F) expression of PD-1 on Siglec-E^+^ or Siglec-E^−^ CD4^+^ T cells from spleen (D), blood (E) and tdLNs (F) as gMFI or percentage positive from each population. G-I) expression of PD-1 on Siglec-E^+^ or Siglec-E^−^ CD8^+^ T cells from spleen (G), blood (H) and tdLNs (I) as gMFI or percentage positive from each population. J-L) CD8 expression on Siglec-E^+^ or Siglec-E^−^ CD8^+^ T cells from spleen (J), blood (K) and tdLNs (L) as gMFI. M,N) expression of Siglec-E on APCs in tdLNs as gMFI (M) or percentage positive from each APC population (N). Data shown as mean ± s.d. from 6 mice. Paired *t-*test for statistical analysis. Ns = *P* > 0.05, ^*^ = *P* < 0.05, ^**^ = *P* < 0.01, ^***^ = *P* < 0.001, ^****^ = *P* < 0.0001. FMO = fluorescence minus one.

T cells are important mediators of anti-tumour immunity, which are primed in secondary lymphoid organs such as lymph nodes and spleen, and then circulate through the blood to reach the TME. As we identified high Siglec-E expression on tumour-infiltrating CD8^+^ T cells, we sought to determine if Siglec-E was also expressed on T cells from spleen, lymph nodes and blood of tumour-bearing mice (Supplementary Fig. S2A-C). In contrast to tumour-infiltrating T cells, we identified Siglec-E on both CD8^+^ and CD4^+^ T cells in spleen, blood and tumour-draining lymph nodes (tdLNs) (Fig. 3A-C). Notably, in spleen and blood CD8^+^ T cells expressed higher Siglec-E than CD4^+^ T cells (Fig. 3A and B), while in tdLNs CD4^+^ T cells expressed slightly higher levels of Siglec-E (Fig. 3C).

Contrary to tumours, in spleen, blood and tdLNs Siglec-E^−^ T cells express lower PD-1 than their Siglec-E^+^ counterparts (Fig. 3D-I). Siglec-E^+^CD8^+^ T cells from spleens expressed higher CD8 than their Siglec-E^−^ counterparts (Fig. 3J), while in blood there was no difference (Fig. 3K), and in tdLNs the relationship was inversed (Fig. 3L). Overall, this suggests that the biology of Siglec-E on T cells and PD-1 co-expression differs across tissues.

### Siglec-E expression on antigen-presenting cells in tumour-draining lymph nodes

Adaptive immune responses require priming of T cells by antigen-presenting cells (APCs). Upon activation, T cells upregulate the expression of Siglec-E ligands which can interact with Siglec-E on APCs suppressing antigen presentation and T cell activation (Redelinghuys et al. 2011; Wang et al. 2022). As the priming of T cells occurs primarily in tdLNs, we explored the expression of Siglec-E on APCs from tdLNs (Supplementary Fig. S2C and D). B cells were the most abundant APCs (Supplementary Fig. S2D) but expressed the lowest Siglec-E levels (Fig. 3M and N). Siglec-E expression in DCs was higher, and similar between conventional DCs type 1 (cDC1) and cDC2 (Fig. 3M and N). Macrophages were scarce, but expressed high levels of Siglec-E (Supplementary Fig. S2D, Fig. 3M and N).

## Discussion

Here, we described the glycosylation of the B16OVA mouse melanoma cell line, identifying GSLs, N- and O-glycans. Using flow cytometry, we determined that this cell line expresses both α2,3-linked and α2,6-linked sialic acids. Further characterization using mass spectrometry-based glycomics revealed that B16OVA cells contain a higher abundance of sialylated GSLs, compared to sialylated N- and O-glycans. GSLs were mostly α2,3-sialylated, while N- and O-glycans contained both α2,3- and α2,6-sialylation. The abundance of α2,3- and α2,6-sialylation in N- and O-glycans was not quantified in this study.

B16OVA sialoglycans interact with Siglec-E. This receptor binds mostly O-glycans on B16OVA cells. Siglec-E is considered a functional paralogue of Siglec-9. Although Siglec-9 has been reported to bind O-glycans on MUC1, it mainly binds N-glycans (Beatson et al. 2016; Narimatsu et al. 2019). Siglec-E is also evolutionary related to Siglec-7 (Angata et al. 2022). Notably, Siglec-7 binds primarily O-glycans, reinforcing the hypothesis that Siglec-E presents characteristics of both Siglec-7 and -9 (Narimatsu et al. 2019; Angata et al. 2022).

In the B16OVA TME, Siglec-E is present on myeloid and lymphoid cells, indicating that these immune cells may be suppressed when interacting with the sialoglycans of B16OVA. Siglec-E was mostly expressed by neutrophils and macrophages in B16OVA tumours, in line with data in other cancers (Ibarlucea-Benitez et al. 2021; Stanczak et al. 2022; Wieboldt et al. 2024). In the TME, Siglec-E was present on CD8^+^ but not CD4^+^ T cells. Siglec-E^+^CD8^+^ T cells were EM and co-expressed low levels of PD-1 and CD8, indicating that they are activated T cells. In contrast to previous data in mouse colorectal cancer, we observed that Siglec-E^+^CD8^+^ T cells expressed lower PD-1 and CD8 than their Siglec-E^−^ counterparts, indicating that Siglec-E^+^CD8^+^ T cells may be more activated and have higher effector potential than their Siglec-E^−^ counterparts. Tumour-infiltrating (EM) CD8^+^ T cells express Siglec-9 in melanoma and lung cancer patients, and are enriched in the TME compared to blood (Stanczak et al. 2018; Haas et al. 2019), similar to our results in mice. Haas et al. observed opposite results in Siglec-9^+^CD8^+^ T cells from melanoma patients, further supporting that Siglec-E may not fully represent Siglec-9.

APCs expressed Siglec-E in both the TME and tdLNs. However, in the TME mostly Siglec-E^+^ macrophages are found, while in the tdLNs there are mostly Siglec-E^+^ DCs. Consequently, the impact of Siglec-E on antigen presentation and T cell priming may also vary according to the APCs present in each tissue context.

Distinct expression levels of Siglec-E and co-expression with PD-1 on T cells were found depending on the tissue examined, suggesting different regulation mechanisms for these immune checkpoints. In contrast to B16OVA tumours, both CD8^+^ and CD4^+^ T cell populations from spleen, blood and tdLNs expressed Siglec-E. Siglec-E^+^CD8^+^ T cells were higher in tumours than in spleen, blood and tdLNs. Our results are in line with data showing that Siglec-E^+^CD8^+^ T cells are higher in tumours than in splenocytes in a mouse colorectal cancer model (Stanczak et al. 2018). This supports the higher immune-suppressive role of Siglec-E in the TME compared to other tissues. Notably, Siglec-E^+^CD8^+^ T cells from spleen, blood and tdLNs had more PD-1 than Siglec-E^−^CD8^+^ T cells. CD8 expression was lower only in Siglec-E^+^ T cells in tdLNs and the TME, suggesting local activation. Overall, our data indicates that the phenotypes of Siglec-E^+^ T cells and the co-expression of this Siglec with PD-1 vary across tissues, suggesting distinct regulatory mechanisms that need to be elucidated and considered, especially when applying combination therapies targeting PD-1 and the sialic acids-Siglec axis.

## Materials and methods

### B16OVA cell line

The B16OVA cell line was obtained from Prof. T.N. Schumacher (Netherlands Cancer Institute) and cultured in RP10 medium [RPMI-1640 medium (Gibco) supplemented with 10% Fetal Calf Serum (Biowest), 2 mM L-glutamine and 1000 U/mL Penicillin–Streptomycin (all Gibco)], and incubated at 37 °C and 5% CO_2_.

### Glycomics

B16OVA cells were harvested and washed 2 times with ice-cold Phosphate Saline Buffer (PBS). The pellet was snap-frozen in dry ice and stored at −70 degrees for subsequent profiling of glycosphingolipids, O-glycans and N-glycans by PGC nano-LC-ESI-MS/MS. Details are in Supplementary Materials and Methods.

### Conventional flow cytometry

B16OVA cells were washed with PBS and stained with Fixable Viability Dye eFluor 450 (Invitrogen) for 10 min. After washing with Hanks' Balanced Salt Solution+0.5% Bovine Serum Albumin (HBSS-BSA), cells were incubated with a mix of SiaFind Pan-Specific, Alpha2,3-Specific or Alpha2,6-Specific biotinylated Lectenz (2ug/mL, LectenzBio) and Streptavidin-APC (Becton Dickinson); or with a mix of Recombinant Mouse Siglec E-Fc Chimera (carrier-free) (Biolegend) and APC anti-human IgG Fc Antibody (Biolegend), both in HBSS-BSA; or a mix of of Anti-Polysialic acid [735], Rabbit IgG, Kappa (Absolute Antibody) and secondary donkey anti-rabbit IgG AlexaFluor647 (Invitrogen) in PBS + 0.5% BSA (PBA), for 30 min. Cells were washed once with PBS and fixated with 2% Paraformaldehyde (PFA) in PBS for 15 min. Cells were acquired in the BD LSRFortessa X-20 cytometer. All incubations were performed at 4 °C in the dark. FlowJo v10.10 was used for data analysis.

### Glycosylation inhibitors

B16OVA cells were plated (150.000 cells/T25 flask) and incubated for three days with culture medium alone or adding dimethyl sulfoxide (DMSO, Carl ROTH), kifunensine (Tocris bioscience, 2ug/mL), benzyl-α-GalNAc (MedChem express, 2 mM) or DL-threo-1-Phenyl-2-palmitoylamino-3-morpholino-1-propanol (PPMP, Enzo life sciences, 1uM).

### In vivo experiments

C57Bl6/J mice were purchased from Charles River and kept at the Animal Research Institute Amsterdam (ARIA) of the Amsterdam UMC (The Netherlands) under pathogen-free conditions. All experiments were approved by the Animal Welfare Body from the Amsterdam UMC (IvD). Subcutaneous injection of B16OVA cells (300.000cells/100uL PBS) was performed under 2%–3% isoflurane.

Tumours were cut into small pieces with sterile scissors in Liberase TL (Roche, 0.75 mg/mL in PBS) and incubated for 25 min at 37 °C shaking. After addition of RP10 HE medium (RP10 medium+10 mM EDTA+20 mM HEPES+20uM 2-mercaptoethanol), 10-minutes incubation, filtering through 70 μm filters, they were spun down and resuspended in RP10 medium.

Spleens were smashed through a 70um filter in RP10 medium+50uM Beta-mercaptoethanol. After spinning down, they were incubated in 1 mL ACK Lysing Buffer (ThermoFisher) at room temperature (RT) for 2–3 min. After adding medium, they were spun down and resuspended in medium.

Whole blood was collected by venipuncture in heparin-treated tubes. Serum was separated by centrifugation at 300×g for 6 min at RT. The cell pellet was washed with PBS at 300×g for 6 min at RT, resuspended in ACK Lysing Buffer (ThermoFisher) for a 3-min incubation at RT, and white blood cells were collected by centrifugation at 300×g for 6 min at RT.

tdLNs were punched with a 27G needle in a polypropylene tube. 0.5 ml of Digestion mix (Liberase TL 100 μg/mL in PBS + 50 μg/mL DNAse, both Roche) was added and incubated in water bath at 37 °C shaking for 30 min. After addition of cold RP10 HE medium, they were passed over a 70um cell strainer. Cells were collected by spinning at 1500 rpm for 10 minutes.

### Spectral flow cytometry

Cells were washed with PBS and stained for 15 min with LIVE/DEAD™ Fixable Blue (Invitrogen). Cells were stained with different antibodies (Supplementary Materials and Methods) in PBA together with True-Stain Monocyte Blocker™ and Purified anti-mouse CD16/32 Antibody (both Biolegend) for 30 min, and fixated with 2% PFA. All incubations were at 4 °C in the dark. Samples were acquired in the 4-Lasers Cytek Aurora™ cytometer. SpectroFlo® and FlowJo v10.10 softwares were used for data analysis.

## Supplementary Material

Final_Supps_all_cwag041

## Data Availability

Data from this manuscript is available upon request from the corresponding author.
