# Supplementary material for: Sialylation profile and Siglec-E expression across tissues in the B16OVA melanoma mouse model
Source: Glycobiology. 2026 Jun 1;36(7):cwag041. doi: 10.1093/glycob/cwag041 (PMC13229241; doi:10.1093/glycob/cwag041)
Supplement: Final_Supps_all_cwag041 [file final_supps_all_cwag041.pdf]

Supplementary Figures

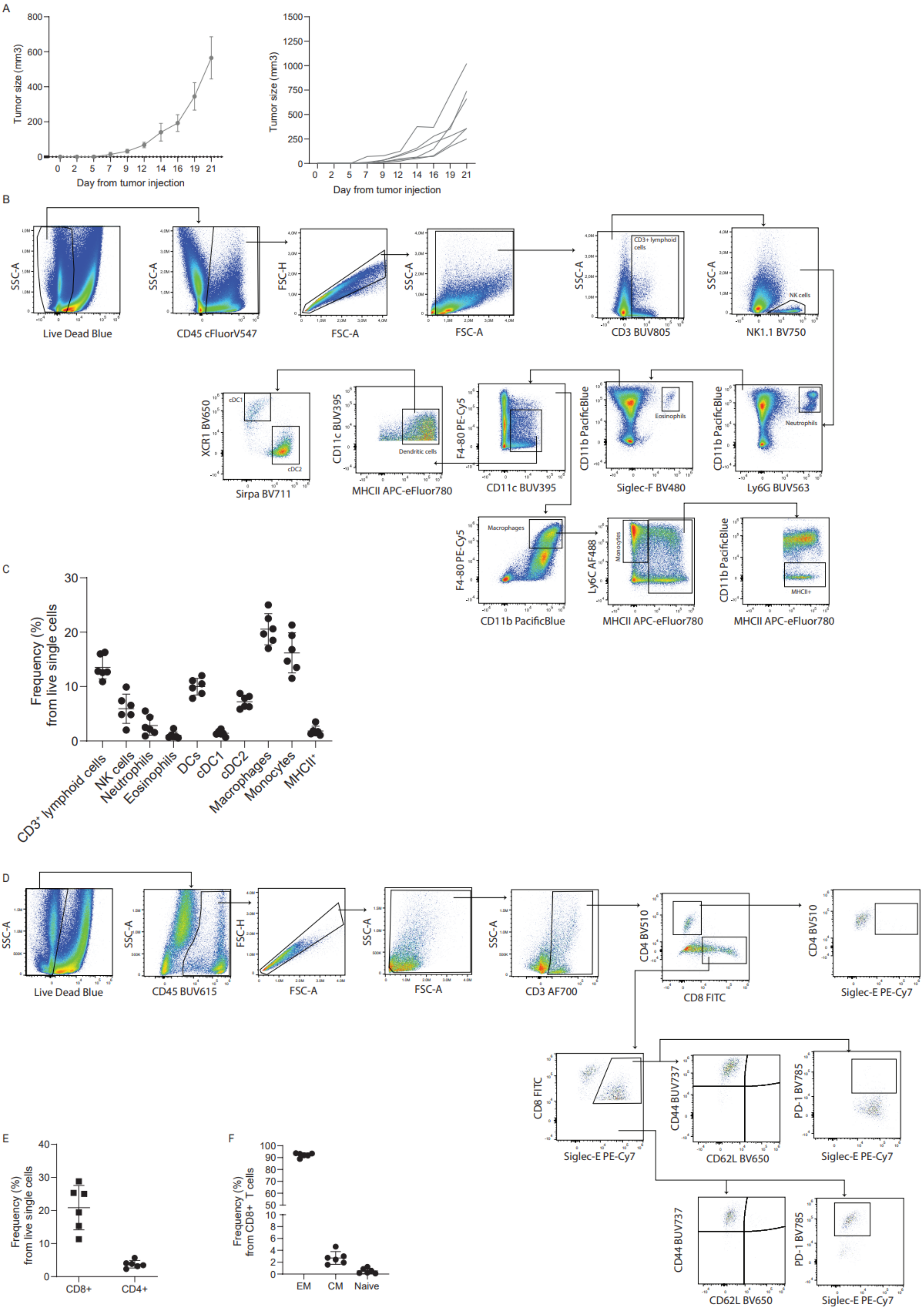

Supplementary Figure S1. B16OVA subcutaneous tumours immune profiling by spectral flow cytometry. A) Tumour size over time from mice injected subcutaneously with B16OVA cells on average (left) or per mouse (right). **B)** Gating strategy to define immune populations from Fig. 1A,B in B16OVA tumours by spectral flow cytometry. **C)** Percentage of immune cell types from Fig. 1A,B in B16OVA subcutaneous tumours. **D)** Gating strategy to define T cell populations from Fig. 1E,F in B16OVA tumours. **E)** Percentage of CD4+ or CD8+ T cells in B16OVA subcutaneous tumours. **F)** Percentage of effector memory (EM), central memory (CM) and naïve CD8+ T cells in B16OVA subcutaneous tumours. Data shown as mean ± s.d. from n=6 mice.

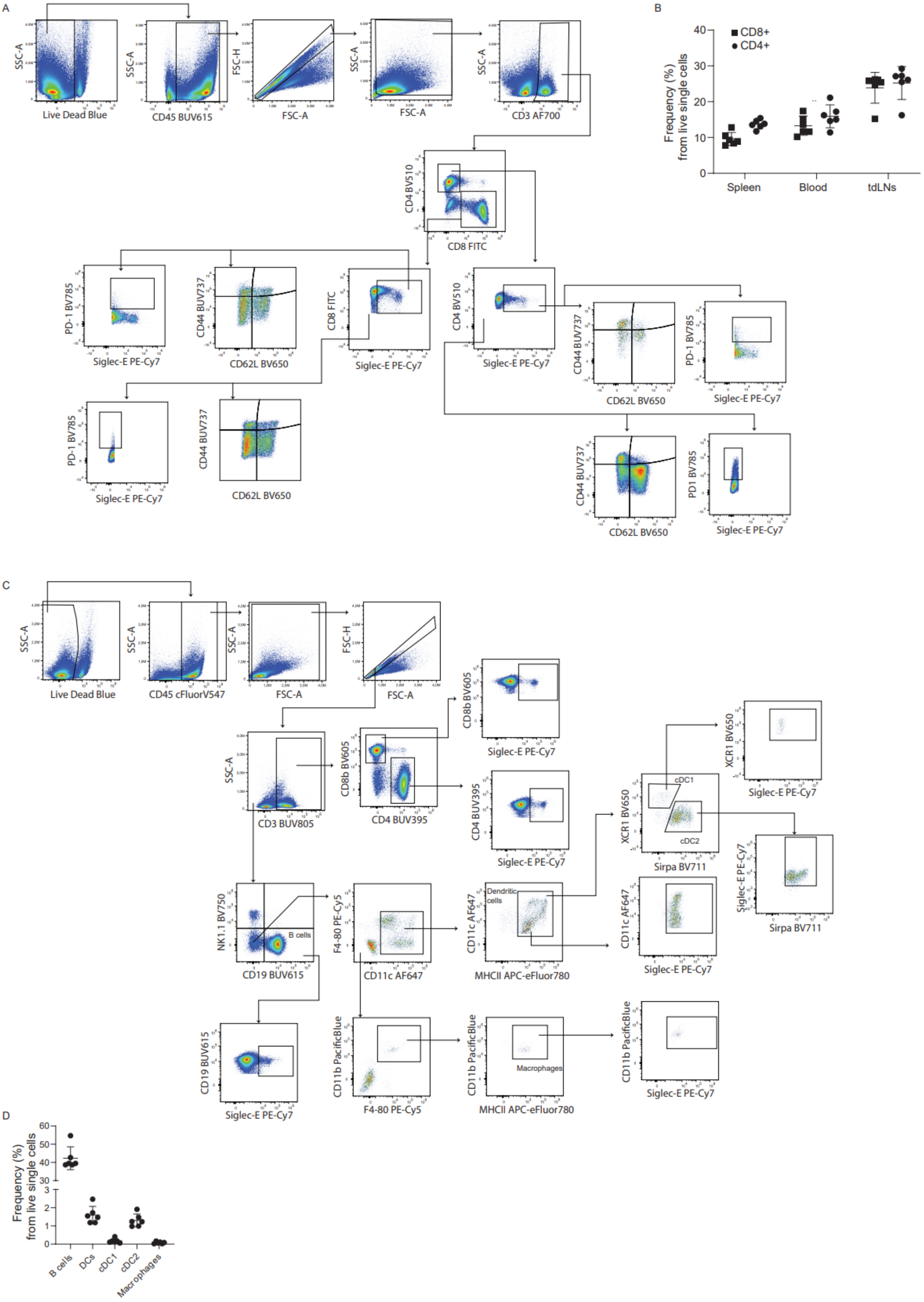
A
SSC-A
Live Dead Blue
CD45 BUV615
FSC-H
FSC-A
CD3 AF700
CD4 BV510
CD8 FITC
PD-1 BV785
Siglec-E PE-Cy7
CD44 BUV737
CD62L BV650
CD8 FITC
PD1 BV785
B
CD8+
CD4+
Frequency (%)
from live single cells
Spleen
Blood
tdLNs
C
CD45 cFluorV547
CD8b BV605
CD3 BUV805
CD4 BUV395
XCR1 BV650
cDC1
cDC2
Sirpa BV711
NK1.1 BV750
B cells
CD19 BUV615
F4-80 PE-Cy5
CD11c AF647
Dendritic cells
MHCII APC-eFluor780
CD11b PacificBlue
Macrophages
D
Frequency (%)
from live single cells
B cells
DCs
cDC1
cDC2
Macrophages

Supplementary Figure S2. Immune profiling of spleen, blood and tdLNs from B16OVA tumour-bearing mice by spectral flow cytometry. A) Gating strategy to define T cell populations in spleen and blood. **B)** Percentage of CD4+ and CD8+ T cells in spleen, blood and tumour-draining lymph nodes (tdLNs). **C)** Gating strategy to define immune populations in tdLNs. **D)** Percentage of antigen-presenting cells in tdLNs. Data shown as mean ± s.d. from n=6 tumour-bearing mice.

Supplemetary Tables

Supplementary Table S1. Relative quantification of GSL-glycan structures assigned based on MS/MS fragmentation and glycobiological pathway constraints. Structures are depicted according to the CFG (Consortium of Functional Glycomics). Blue square: *N*-acetylglucosamine, yellow square: *N*-acetylgalactosamine, blue circle: glucose, yellow circle: galactose, red triangle: fucose, pink diamond: *N*-acetylneuraminic acid, white diamond: *N*-glycolylneuraminic acid. a, b: isomer number; SD: standard variation

Glycan number	Glycan name	Proposed structure	Relative abundance %. (SD %) WT	Theoretical $[M-H]^-$	Observed $[M-H]^-$	Deviation $\Delta[M-H]^-$
1	Lc2	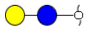	0.031 (±0.002)	343.125	343.123	0.002
2	Gb3	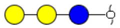	1.999 (±0.114)	505.177	505.172	0.005
3	Gb4	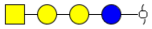	0.004 (±0.001)	708.257	708.250	0.007
4	SSEA3	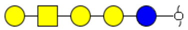	0.035 (±0.002)	870.310	870.298	0.012
5	GM3	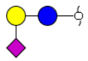	0.016 (±0.004)	634.220	634.213	0.007
6	GM3-Neu5Gc	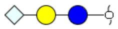	0.017 (±0.005)	650.215	650.207	0.008
7	GM1a	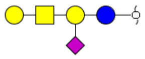	0.054 (±0.004)	999.352	999.336	0.016
8	GD1a	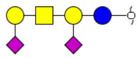	96.962 (±0.120)	1290.448	1290.427	0.021
9	GD3	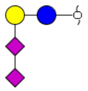	0.701 (±0.005)	925.315	925.303	0.012

**10**	**Gg4**	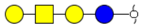	**0.059 (±0.002)**	**708.257**	**708.248**	**0.009**
**11**	**Gal-Gg4**	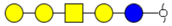	**0.007 (±0.001)**	**870.310**	**870.297**	**0.013**
**12**	**Lc3**	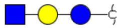	**0.004 (±0.001)**	**546.204**	**546.198**	**0.006**
**13**	**nLc4**	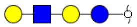	**0.020 (±0.001)**	**708.257**	**708.246**	**0.011**
**14**	**S(6)nLc4**	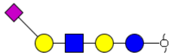	**0.012 (±0.001)**	**999.352**	**999.336**	**0.016**
**15**	**S(3)nLc4**	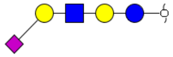	**0.057 (±0.001)**	**999.352**	**999.336**	**0.016**
**16**	**S(3)nLc4-Neu5Gc**	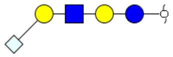	**0.004 (±0.001)**	**1015.347**	**1015.326**	**0.021**
**17**	**S(3)nLc6**	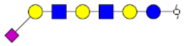	**0.020 (±0.003)**	**1364.484**	**1364.484**	**0.000**

Supplementary Table S2. Relative quantification of O-Glycan structures assigned based on MS/MS fragmentation and glycobiological pathway constraints. Structures are depicted according to the CFG (Consortium of Functional Glycomics). Blue square: *N*-acetylglucosamine, yellow square: *N*-acetylgalactosamine, blue circle: glucose, yellow circle: galactose, red triangle: fucose, pink diamond: *N*-acetylneuraminic acid, white diamond: *N*-glycolylneuraminic acid, S: sulfate. a, b: isomer number; SD: standard variation.

Glycan number	Glycan composition	Proposed structure	Relative abundance %. (SD %) WT	Theoretical $[M-H]^-$	Observed $\Delta[M-H]^-$	Deviation $\Delta[M-H]^-$
1	N1H1	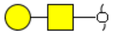	0.031 (±0.032)	384.151	384.149	0.002
2	N1S1	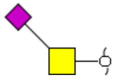	1.884 (±0.148)	513.194	513.189	0.005
3	N1H1S1a	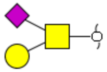	3.498 (±0.177)	675.247	675.241	0.006
4	N1H1S1b	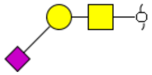	14.370 (±0.201)	675.247	675.240	0.007
5	N1H1S2	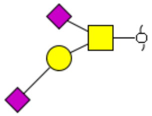	58.100 (±1.154)	966.342	966.332	0.010
6	N2H1	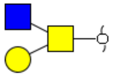	0.466 (±0.185)	587.231	587.225	0.006
7	N2H2a	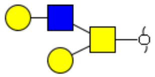	0.192 (±0.048)	749.283	749.277	0.006

8	**N2H2b**	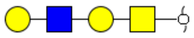	**0.222 (±0.091)**	**749.283**	**749.210**	**0.063**
9	**N2H2F1**	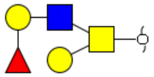	**0.633 (±0.104)**	**895.341**	**895.333**	**0.008**
10	**N2H2F3**	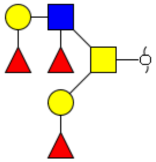	**1.800 (±0.140)**	**1187.457**	**1187.409**	**0.048**
11	**N2H2S2**	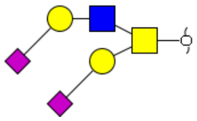	**11.139 (±0.483)**	**1331.474**	**1331.469**	**0.005**
12	**N2H2F1S2**	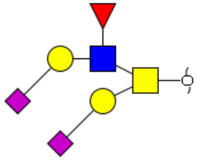	**0.999 (±0.403)**	**1477.532**	**1447.464**	**0.068**
13	**N3H1**	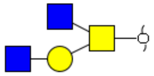	**3.112 (±0.220)**	**790.310**	**790.253**	**0.057**
14	**N3H3**	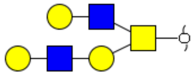	**0.340 (±0.041)**	**1114.416**	**1114.434**	**0.022**
15	**N3H3F1S1Su1**	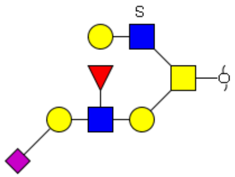	**2.897 (±0.066)**	**1631.526**	**1631.610**	**0.084**

Supplementary Table S3. Relative quantification of N-Glycan structures assigned based on MS/MS fragmentation and glycobiological pathway constraints. Structures are depicted according to the CFG (Consortium of Functional Glycomics). Blue square: *N*-acetylglucosamine, yellow square: *N*-acetylgalactosamine, blue circle: glucose, yellow circle: galactose, red triangle: fucose, pink diamond: *N*-acetylneuraminic acid, white diamond: *N*-glycolylneuraminic acid, P, phosphate. a, b, c: isomer number; SD: standard variation.

Glycan number	Glycan composition	Proposed structure	Relative abundance %. (SD %)	Theoretical	Observed	Deviation
			WT	$[M-H]^-$	$[M-H]^-$	$\Delta[M-H]^-$
1	H2N2	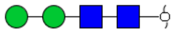	0.528 (±0.103)	749.283	749.274	0.009
2	H2N2F1	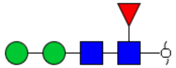	0.827 (±0.086)	895.341	895.329	0.012
3	H3N2	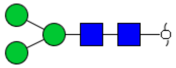	0.003 (±0.002)	911.336	911.324	0.012
4	H3N2P1	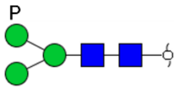	0.007 (±0.002)	991.303	991.298	0.005
5	H3N2F1	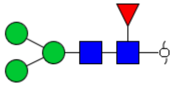	0.930 (±0.025)	1057.394	1057.379	0.015
6	H4N2	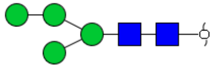	0.226 (±0.027)	1073.389	1073.382	0.007

**7**	**H4N2F1**	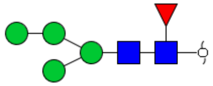	**0.017(±0.001)**	**1219.447**	**1219.441**	**0.006**
**8**	**H5N2**	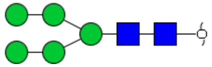	**1.931 (±0.267)**	**1235.442**	**1235.425**	**0.017**
**9**	**H6N2**	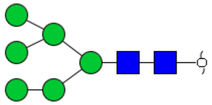	**8.872 (±0.582)**	**1397.495**	**1397.479**	**0.016**
**10**	**H6N2P1**	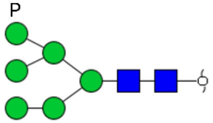	**1.593 (±0.013)**	**1477.461**	**1477.442**	**0.019**
**11**	**H7N2**	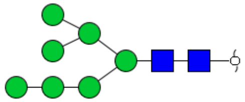	**6.028 (±0.149)**	**1559.547**	**1559.529**	**0.018**
**12**	**H7N2P1**	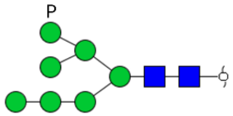	**1.479 (±0.068)**	**1639.514**	**1639.489**	**0.025**
**13**	**H7N2P2**	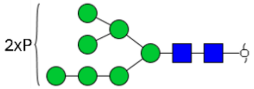	**0.482 (±0.056)**	**1719.480**	**1719.398**	**0.082**
**14**	**H8N2**	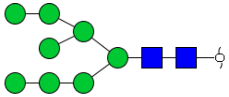	**11.983 (±0.414)**	**1721.600**	**1721.581**	**0.019**
**15**	**H8N2P1**	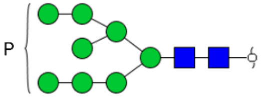	**0.424 (±0.047)**	**1801.567**	**1801.558**	**0.009**

16	H9N2	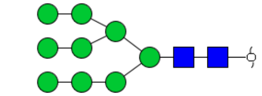	14.622 (±0.452)	1883.653	1883.632	0.021
17	H9N2P1	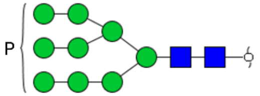	0.263 (±0.051)	1963.619	1963.601	0.018
18	H10N2	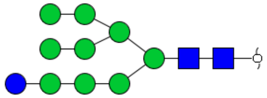	1.810 (±0.167)	2045.706	2045.672	0.034
19	H3N3	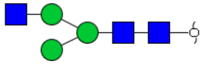	0.064 (±0.016)	1114.416	1114.406	0.010
20	H3N3F1	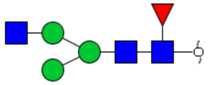	0.105 (±0.019)	1260.473	1260.461	0.012
21	H4N3S1	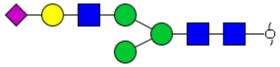	0.186 (±0.087)	1567.564	1567.542	0.022
22	H4N3F1S1	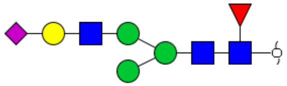	0.546 (±0.191)	1713.622	1713.582	0.040
23	H5N3F1	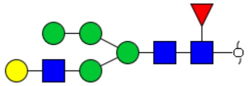	0.081 (±0.026)	1584.579	1584.566	0.013
24	H5N3S1	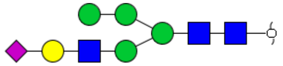	0.247 (±0.043)	1729.617	1729.609	0.008
25	H5N3F1S1a	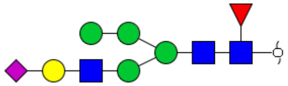	0.479 (±0.083)	1875.675	1875.640	0.035

26	H5N3F1S1b	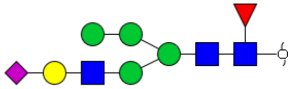	0.410 (±0.049)	1875.675	1875.642	0.033
27	H6N3	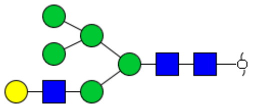	0.528 (±0.119)	1600.574	1600.568	0.006
28	H6N3F1a	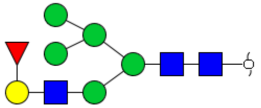	0.507 (±0.062)	1746.632	1746.628	0.004
29	H6N3F1b	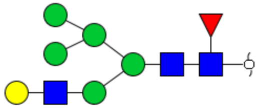	0.165 (±0.073)	1746.632	1746.626	0.006
30	H6N3S1a	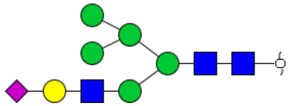	1.021 (±0.159)	1891.669	1891.637	0.032
31	H6N3S1b	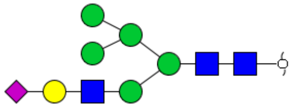	0.985 (±0.149)	1891.669	1891.635	0.034
32	H6N3F1S1a	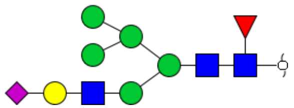	0.398 (±0.072)	2037.727	2037.693	0.034
33	H6N3F1S1b	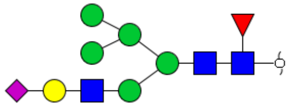	0.208 (±0.038)	2037.727	2037.693	0.034
34	H7N3F1a	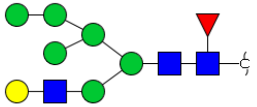	0.659 (±0.070)	1908.685	1908.678	0.007

35	H7N3F1b	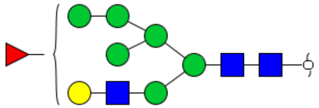	0.129 (±0.092)	1908.685	1908.676	0.009
36	H3N4	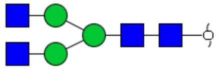	0.085 (±0.007)	1317.495	1317.486	0.009
37	H3N4F1	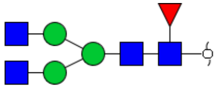	0.335 (±0.039)	1463.553	1463.533	0.020
38	H4N4F1	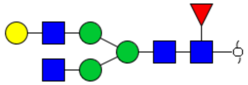	0.306 (±0.076)	1625.606	1625.577	0.029
39	H4N4F1S1	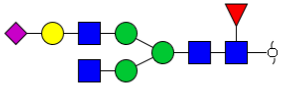	0.468 (±0.063)	1916.701	1916.667	0.034
40	H5N4	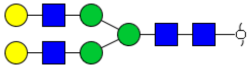	0.161 (±0.133)	1641.601	1641.568	0.033
41	H5N4F1	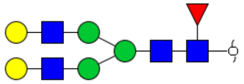	0.501 (±0.336)	1787.659	1787.626	0.033
42	H5N4S1a	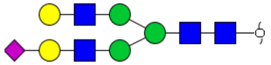	1.102 (±0.269)	1932.696	1932.662	0.034
43	H5N4S1b	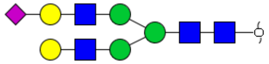	0.935 (±0.286)	1932.696	1932.661	0.035
44	H5N4F1S1a	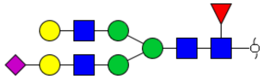	1.078 (±1.295)	2078.754	2078.719	0.034

45	H5N4F1S1b	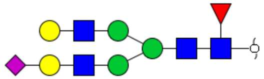	1.413 (±0.784)	2078.754	2078.720	0.034
46	H5N4S2a	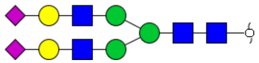	0.762 (±0.114)	2223.791	2223.753	0.038
47	H5N4S2b	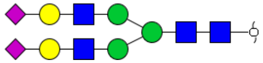	1.748 (±0.418)	2223.791	2223.754	0.037
48	H5N4S2c	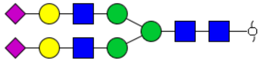	0.569 (±0.145)	2223.791	2223.754	0.037
49	H5N4F1S2a	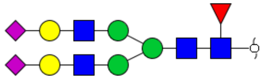	2.133 (±0.315)	2369.849	2369.819	0.030
50	H5N4F1S2b	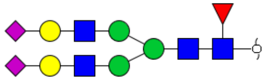	4.315 (±0.1.020)	2369.849	2369.811	0.038
51	H5N4F1S2c	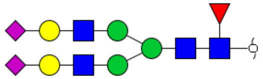	0.974 (±0.340)	2369.849	2369.810	0.039
52	H5N5S1a	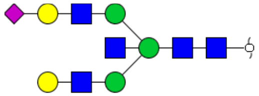	0.156 (±0.008)	2135.775	2135.737	0.038
53	H5N5S1b	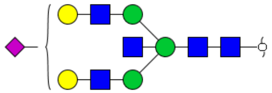	0.095 (±0.029)	2135.775	2135.738	0.037
54	H5N5S2	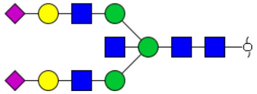	0.265 (±0.085)	2426.871	2426.829	0.042

55	H6N5F1a	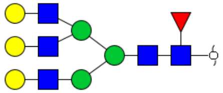	0.074 (±0.043)	2152.791	2152.752	0.039
56	H6N5F1b	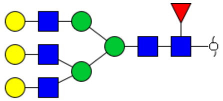	0.074 (±0.042)	2152.791	2152.749	0.042
57	H6N5F1S1a	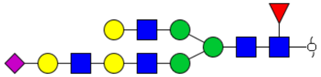	0.134 (±0.071)	2443.886	2443.841	0.045
58	H6N5F1S1b	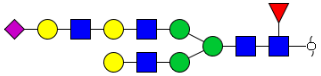	0.228 (±0.108)	2443.886	2443.842	0.044
59	H6N5F1S1c	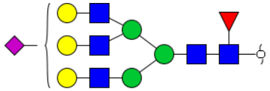	0.176 (±0.107)	2443.886	2443.842	0.044
60	H5N5F1S2	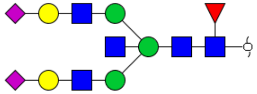	1.440 (±0.688)	2572.929	2572.890	0.039
61	H6N5F1S2	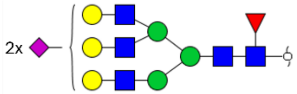	5.802 (±2.619)	2734.982	2734.749	0.033
62	H6N5S3	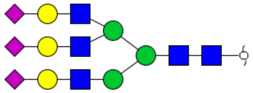	1.124 (±0.211)	2880.019	2879.970	0.049
63	H6N5F1S3a	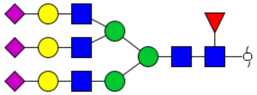	0.602 (±0.243)	3026.077	3026.025	0.052

64	H6N5F1S3b	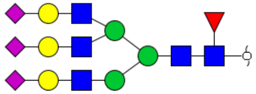	2.688 (±0.936)	3026.077	3026.027	0.050
65	H7N6F1	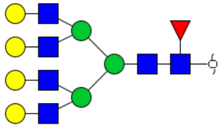	0.084 (±0.020)	2517.923	2517.892	0.031
66	H7N6F1S2	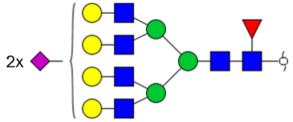	1.554 (±0.511)	3100.114	3100.056	0.058
67	H7N6F1S3	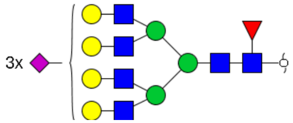	3.095 (±0.660)	3391.209	3391.137	0.072
68	H7N6S4	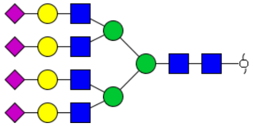	0.916 (±0.469)	3536.247	3536.194	0.053
69	H7N6F1S4	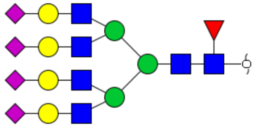	5.865 (±3.977)	3682.305	3682.251	0.054

Supplementary Materials and Methods

Mass spectrometry-based glycomics analysis

Glacial acetic acid, formic acid (FA), 2-propanol and potassium hydroxide (KOH) were purchased from Honeywell Fluka. Ammonium bicarbonate (ABC), sodium borohydride (NaBH4), cation-exchange resin Dowex (50W-X8), DLdithiothreitol (DTT) and hydrochloric acid (HCl) were obtained from Sigma-Aldrich (Germany). Peptide Nglycosidase F (PNGase F) was obtained from Roche Diagnostics (Germany). 8M guanidine hydrochloride (GuHCl) was obtained from Thermo Fisher Scientific (USA). MultiScreen HTS 96-well plates (hydrophobic Immobilon-P PVDF membrane) were obtained from Millipore (the Netherlands) and 96-well PP filter plate from Orochem Technologies (USA). Bulk sorbent Carbograph was obtained from Grace Discovery Sciences (USA). Acetonitrile and methanol were purchased from Actu-All Chemicals (the Netherlands). Ultrapure water was generated from an ELGA Labwater system (the Netherlands).

GSL-, N- and O-glycan alditols released from $0.5x10^6$ cells were prepared in 96-well plate format as described previously with slight modification (Zhang et al., 2020; Zhang et al., 2024). After sample denaturation and cleanup, GSL-glycans were firstly released from sample followed by reduction, desalting and PGC SPE cleanup. Prior to GSL extraction, the samples were spiked with 10 μL of 0.5 μM isoglobotriosylceramide (C17:0 iGb3; Avanti Polar Lipids) in ethanol, which was employed for normalization and semi-absolute quantification of the GSL-glycans. Next, N- and O-glycans were released from the sample using enzymatic digestion and beta-elimination, spiked with maltoheptaose (DP7, 5 ng; Sigma-Aldrich) and maltopentaose (DP5, 5 ng; Sigma-Aldrich) during the release of N- and O-linked glycans, respectively. The spiked glycans were employed as internal

standard for normalization and semi-absolute quantification of the respective glycan species.

Glycan alditols were redissolved in 10µL of water prior to porous graphitized carbon nano-liquid chromatography (PGC nano-LC-ESIMS/MS) analysis. The LC-MS setup consisted of a home-packed PGC trap column (5µm Hypercarb, 320µm x 30mm) and PGC nano-column (3µm Hypercarb 100µm x 150mm) connected to a timsTOF fleX MALDI-2 instrument employing a nano ESI source (Bruker Daltonics, Germany). The temperature of the column was maintained at 35°C. Ionization was achieved using the nanoBooster source (Bruker) with a capillary voltage of 1000V applied in negative ion mode with isopropanol-enriched dopant nitrogen. MS spectra were acquired with an *m/z* range of 150-3000. MS/MS spectra were generated using collision-induced dissociation over an *m/z* range from 150 to 3000 on the top four most abundant precursors. Glycan structures were assigned based on their known MS/MS fragmentation patterns in negative-ion mode (Karlsson et al., 2004) , elution order, and general glycobiological knowledge, with help of Glycoworkbench(Ceroni et al., 2008) and Glycomod(Cooper et al., 2001) software. Relative quantification of individual glycans was performed by normalizing the total peak area of all glycans within one sample to 100%. To estimate the glycan molecule number per cell, individual glycan intensity was normalized to the intensity of the internal standard, assuming complete release of all glycans and similar MS response factors between the released glycans and the spiked standard.

Spectral flow cytometry antibodies

Different antibodies were used in different panels for the staining of B16OVA tumours, spleen, blood and tumour-draining lymph nodes which can be found in the following table:

Antibody	Fluorochrome	Company	Catalogue
Anti-Mouse CD45 (30-F11)	cFluorV547	Cytek	SKU R7-20571
BD OptiBuild™ Hamster Anti-Mouse CD3	BUV805	Becton Dickinson	749276
BD Horizon™ Hamster Anti-Mouse CD11c	BUV395	Becton Dickinson	564080
BD OptiBuild™ Mouse Anti-Mouse NK-1.1	BV750	Becton Dickinson	746876
Anti-mouse/human CD11b Antibody	Pacific Blue	Biolegend	101224
MHC Class II (I-A/I-E) Monoclonal Antibody (M5/114.15.2)	APC-eFluor780	Invitrogen	47-5321-82
Anti-mouse/rat XCR1 Antibody	BV650	Biolegend	148220
BD OptiBuild™ Rat Anti-Mouse CD172a	BV711	Becton Dickinson	740766
Anti-mouse Ly-6C Antibody	AF488	Biolegend	128022
F4/80 Monoclonal Antibody (BM8)	PE-Cy5	Invitrogen	15-4801-82
BD Horizon™ Rat Anti-Mouse Ly-6G	BUV563	Becton Dickinson	612921
Anti-Siglec-E Antibody	PE-Cy7	Biolegend	677108
CD8b Monoclonal Antibody (eBioH35-17.2 (H35-17.2))	FITC	Invitrogen	11-0083-82
Anti-mouse CD3 Antibody	AF700	Biolegend	100216
Anti-mouse CD4 Antibody	BV510	Biolegend	100449
Anti-mouse CD279 (PD-1) Antibody	BV785	Biolegend	135225
Rat Anti-Mouse CD44	BUV737	Becton Dickinson	560569
Anti-mouse CD62L Antibody	BV650	Biolegend	104453
CD45 Monoclonal Antibody (30-F11)	BUV615	Invitrogen	366-0451-80
Rat Anti-Mouse CD19	BUV615	Becton Dickinson	751213
BD Horizon™ Rat Anti-Mouse CD4	BUV395	Becton Dickinson	565974
BD OptiBuild™ Rat Anti-Mouse CD8b	BV605	Becton Dickinson	740387
Anti-mouse CD279 (PD-1) Antibody	BV421	Biolegend	135221
Anti-mouse CD11c Antibody	AF647	Biolegend	117312

References

Ceroni, A., Maass, K., et al. (2008). GlycoWorkbench: A Tool for the Computer-Assisted Annotation of Mass Spectra of Glycans. *Journal of Proteome Research, 7*(4), 1650–1659. doi:10.1021/pr7008252

Cooper, C. A., Gasteiger, E., et al. (2001). GlycoMod--a software tool for determining glycosylation compositions from mass spectrometric data. *Proteomics, 1*(2), 340–349. doi:10.1002/1615-9861(200102)1:2<340::AID-PROT340>3.0.CO;2-B

Karlsson, N. G., Wilson, N. L., et al. (2004). Negative ion graphitised carbon nano-liquid chromatography/mass spectrometry increases sensitivity for glycoprotein

oligosaccharide analysis. *Rapid Commun Mass Spectrom, 18*(19), 2282–2292. doi:10.1002/rcm.1626

Zhang, T., Madunic, K., et al. (2020). Development of a 96-well plate sample preparation method for integrated N- and O-glycomics using porous graphitized carbon liquid chromatography-mass spectrometry. *Mol Omics, 16*(4), 355–363. doi:10.1039/c9mo00180h

Zhang, T., Wang, W., et al. (2024). Comprehensive O-Glycan Analysis by Porous Graphitized Carbon Nanoliquid Chromatography-Mass Spectrometry. *Anal Chem, 96*(22), 8942–8948. doi:10.1021/acs.analchem.3c05826
